# Impact of probiotics on pathogen survival in an innovative human plasma biofilm model (*hp*BIOM)

**DOI:** 10.1186/s12967-019-1990-4

**Published:** 2019-07-25

**Authors:** M. Besser, J. Terberger, L. Weber, B. Ghebremedhin, E. A. Naumova, W. H. Arnold, E. K. Stuermer

**Affiliations:** 1 Institute of Translational Wound Research, Centre for Biomedical Education and Research (ZBAF), Witten/Herdecke University, Stockumer Street 10, 58453 Witten, Germany; 2 Institute for Medical Laboratory Diagnostics, Centre for Clinical and Translational Research (CCTR), HELIOS University Hospital Wuppertal, Wuppertal, Germany; 3 Department of Biological and Material Sciences in Dentistry, School of Dentistry, Faculty of Health, Witten/Herdecke University, Witten, Germany; 4 Institute for Health Care Research in Dermatology and Nursing, University Medical Center Hamburg-Eppendorf (UKE), Hamburg, Germany

**Keywords:** Human plasma biofilm model, hpBIOM, Chronic wounds, Bacteriotherapy, Probiotics, *L. plantarum*, *B. lactis*, *S. cerevisiae*

## Abstract

**Background:**

Despite of medical advances, the number of patients suffering on non-healing chronic wounds is still increasing. This fact is attended by physical and emotional distress and an economic load. The majority of chronic wounds are infected of harmful microbials in a protecting extracellular matrix. These biofilms inhibit wound healing. Biofilm-growing bacteria developed unique survival properties, which still challenge the appropriate wound therapy. The present in-vitro biofilm models are not suitable for translational research. By means of a novel in-vivo like human plasma biofilm model (hpBIOM), this study systematically analysed the influence of 3 probiotics on the survival of five clinically relevant pathogenic microorganisms.

**Methods:**

Human plasma was used to produce the innovate biofilm. Pathogenic microorganisms were administered to the plasma. By stimulating the production of a fibrin scaffold, stable coagula-like discs with integrated pathogens were produced. The five clinically relevant pathogens *P. aeruginosa*, *S. aureus*, *S. epidermidis*, *E. faecium* and *C. albicans* were challenged to the probiotics *L. plantarum*, *B. lactis* and *S. cerevisiae*. The probiotics were administered on top of the biofilm and the survival was quantified after 4 h and 24 h of incubation. For statistics, two-way ANOVA with post-hoc Tukey’s HSD test was applied. P-value > 0.05 was considered to be significant.

**Results:**

SEM micrographs depicted the pathogens on the surface of the fibrin scaffold, arranged in close proximity and produced the glycocalyx. The application of probiotics induced different growth-reducing capacities towards the pathogens. *B. lactis* and *S. cerevisiae* showed slight bacteria-reducing properties. The survival of *C. albicans* was not affected at all. The most antimicrobial activity was detected after the treatment with *L. plantarum*.

**Conclusions:**

This study successfully reproduced a novel human biofilm model, which provides a human wound milieu and individual immune competence. The success of bacteriotherapy is dependent on the strain combination, the number of probiotics and the activity of the immune cells. The eradicating effect of *L. plantarum* on *P. aeruginosa* should be emphasized.

## Background

The demographic change is attended by an increased incidence of the development of chronic wounds leading to a significant socio-economic burden. If a wound displays no signs of healing after 4 weeks, despite of appropriate wound management, it is defined to be a chronic wound. Most patients with chronic wounds suffer from basic diseases that inhibit the perfusion of the tissue, e.g. diabetes, immobility or peripheral vascular insufficiencies. These wounds are from the beginning considered to become a chronic state [1].

The development and progressive maturation initiates with the adhesion and the attachment of bacteria and fungi preferentially on damaged tissue, followed by colonisation and infection via proliferation [2, 3]. The surrounding milieu will be re-organized to a protecting extracellular polymeric substance (EPS) by the microorganisms. It is postulated, that 60–80% of non-healing wounds are challenged to these biofilms [4–6]. The dimension with regard to thickness and diameter varies from some micrometres to a few millimetres [7]. Persistent biofilms affect the well-orchestrated tissue repair process, leading to a prolonged inflammatory phase and, concomitantly, a delayed wound healing. Only 6% of acute wounds are affected by biofilms [4].

Bacteria in biofilms are at least tenfold more resistant to systemically as well as topically applied antibiotics than their planktonic variants, because of their improved survival mechanisms [8, 9]. They show slower growth rate, are able to transfer genes mediating resistances to antibiotics and, due to the EPS, the penetration of antibiotics and other biocides is aggravated [10–12]. During wound therapy, patients are subjected to surgical interventions to destroy the biofilm matrix for cleansing of the wound area. Beside the painful procedure, tissue debridement consequently leads to an increased wound area but success often fails [1, 13]. The most common strains in post-operative wound infections are *Pseudomonas aeruginosa*, *Enterococcus* *faecium* and *Staphylococcus* *aureus* [9]. The importance of finding new treatment methods for wound infections is underlined by the fact, that the recently published WHO recommendation list for R&D of new antibiotics contains more than five wound associated pathogens. The investigated strains in this study are all ranked high or critical priority [14, 15].

In summary, wound healing is impaired by biofilms and successful strategies overcoming this challenge in the wound management are missing.

Alternative therapeutic approaches have to be assigned, one could be the bacteriotherapy. Bacteriotherapy involves the application of non-pathogenic microorganisms (probiotics) to combat the pathogenic biofilm-residing microbials. Probiotics were defined by the Food and Agriculture Organization of the United Nations (FAO) and World Health Organization (WHO) as “live microorganisms, which when administered in adequate amounts, confer a health benefit on the host” [16]. Some studies already proposed a supportive role of probiotics in wound healing, e.g. of burns wounds infected with *Pseudomonas aeruginosa* in mice and rabbits [17, 18]. Recent studies postulated chronic wound pathology may be a result of a dysbiosis of the skin microbiome [19]. Several hypotheses regarding the underlying mechanisms of the therapeutic effects were discussed. Pathogenic microbials can be repressed by natural selection in the competition for micronutrients and a modulation of the host immune system by probiotic bacteria is likely an important factor for the effectiveness of bacteriotherapy [20]. Additionally, some commensals from the naturally occurring human microbiome produce antimicrobial and bactericide substances [21–23]. Nevertheless, knowledge rendering targeted, secure application of bacteriotherapy, to treat human biofilm-challenged chronic wounds is still inaccurate and must be faced in further studies.

In this study, a novel human plasma biofilm model was used to mimic a biofilm-infected human wound environment, to analyse the efficiency of bacteriotherapy regarding the disruption of the EPS and elimination of biofilm-growing pathogenic microorganisms. Five clinically relevant pathogens *Staphylococcus aureus*, *Pseudomonas aeruginosa*, *Staphylococcus epidermidis*, *Enterococcus faecium* and *Candida albicans* were challenged to the lactic-acid producing probiotics *Lactobacillus plantarum* and *Bifidobacterium lactis* and to the human commensal *Saccharomyces cerevisiae*.

## Methods

### Bacteria strains

All strains were obtained from the Leibniz-Institute DSMZ-German Collection of Microorganisms and Cell Cultures. Details are given in Table 1.

**Table 1:** Applied pathogenic and probiotic bacteria

Strain	DSM no	Culture condition: medium, temperature
*Staphylococcus aureus subsp. aureus*	799	CSB, 37 °C
*Staphylococcus epidermidids*	20,044	CSB, 37 °C
*Pseudomonas aeruginosa*	939	CSB, 37 °C
*Enterococcus faecium*	2146	CSB, 37 °C
*Candida albicans*	1386	MEB, 37 °C
*Lactobacillus plantarum subsp. plantarum*	2601	MRS, 37 °C
*Bifidobacterium animalis subsp. lactis*	10,140	CSB, 37 °C
*Saccharomyces cerevisiae*	70,449	MEB, 37 °C

## Experimental setup

### Human plasma biofilm model preparation

Plasma preserves and buffy coats from anonymous donors were obtained from the DRK-Blutspendedienst West (Hagen, Germany) and biofilm model were constructed as described previously [24]. In brief, residual erythrocytes in the buffy coat were removed by a centrifugation for 30 min at 3000 rpm at room temperature (RT). The plasma preserve and the buffy coat were fused and collected in a sterile glass bottle. The content of the bottle was gently mixed and continuously shaked at 22 °C.

A single hpBIOM was comprised of 1.5 ml plasma solution. 1*10^6^ cfu/1.5 ml pathogens were supplemented. 18.26 µl CaCl_2_ (500 mM) per ml plasma were applied, gently mixed and quickly transferred into wells of 12-well culture plates (Sarstedt AG & Co., Nürnbrecht, Germany). The plates were incubated for 1 h on a rotation shaker at 50 rpm and 37 °C. During this time, the plasma polymerized and a stable biofilm disc/clot with integrated pathogens was generated and could be used for further analyzes.

### Administration of probiotics

*L. plantarum*, *B. lactis* and *S. cerevisiae* were grown in MRS, CSB or MEB medium for 2 days at 37 °C with shaking at 50–100 rpm. The probiotics were diluted to provide 1*10^9^ cfu in a maximum volume of 100 µl medium. This concentration was applied on top of the biofilms, followed by an additional dose of 1*10^9^ cfu after 2 h of incubation. Each pathogen was challenged to one single probiotic strain.

### Dissolving of the biofilm and quantification of the bacterial growth

2 h or 24 h after the second application of probiotics, the biofilm models were dissolved by the incubation with 1.5 ml (1:1 v/v) 10% (w/v) bromelain solution (Bromelain-POS^®^, RSAPHARM Arzneimittel GmbH, Saarbrücken, Germany) in 100 ml phosphate buffered saline (PBS). By using a pipette tip, the discs were detached off the well margins and subsequently punctured to make the models more permeable for the enzymatic digestion. After 2 h, the biofilm models were completely dissolved. For the quantification of the potentially survived pathogenic bacteria, 100 µl aliquots from different dilution preparations were streaked out on CSA, MEA or MRS agar plates. The bacterial burden (cfu/ml) was determined by counting colonies with a Colony Counter Pen (eCount™, VWR Leicestershire, UK) after incubation over night at 37 °C.

### Scanning electron microscopy (SEM)

Scanning electron microscopy (SEM) was used to analyse the bacterial morphology. The coagula were fixed with 0.1 M cacodylate buffer containing 2.5% glutaraldehyde, 2% polyvinylpyrrolidone and 75 mM NaNO_2_ for 1 h at 4 °C. Samples were washed in 0.1 M cacodylate buffer without glutaraldehyde and subsequently incubated in a solution containing 2% arginine-HCl, glycine, sucrose and sodium glutamate for 18 h at RT. The specimens were rinsed in distilled water followed by immersion in a mixture of each 2% tannic acid and guanidine-HCl for 5.5 h at RT. The samples were rinsed again in distilled water and incubated in a 1% OsO_4_ solution for 30 min at RT. After three rinsing steps with distilled water the specimens were dehydrated, dried in liquid CO_2_, sputtered with gold palladium and finally examined with a Zeiss Sigma SEM (Zeiss, Oberkochen, Germany) using 2 kV acceleration voltage and an inlens detector.

### Statistical analysis

The experiments were performed in triplicates per donor for each pathogen/probiotic strain combination. Experimental data were analyzed by the statistic package GraphPadPrism 6 (GraphPad Software, Inc., La Jolla, USA). Data are presented as means ± standard deviation (SD). Statistical analysis was performed by applying two-way ANOVA, followed by Tukey´s HSD test as post-hoc evaluation of multiple comparisons. A p-value of *p* ≤ 0.05 was considered significant. (*p ≤ 0.05; **p ≤ 0.01; ***p ≤ 0.001).

## Results

### Reproduction of the novel human plasma biofilm model (hpBIOM)

**Fig. 1:**
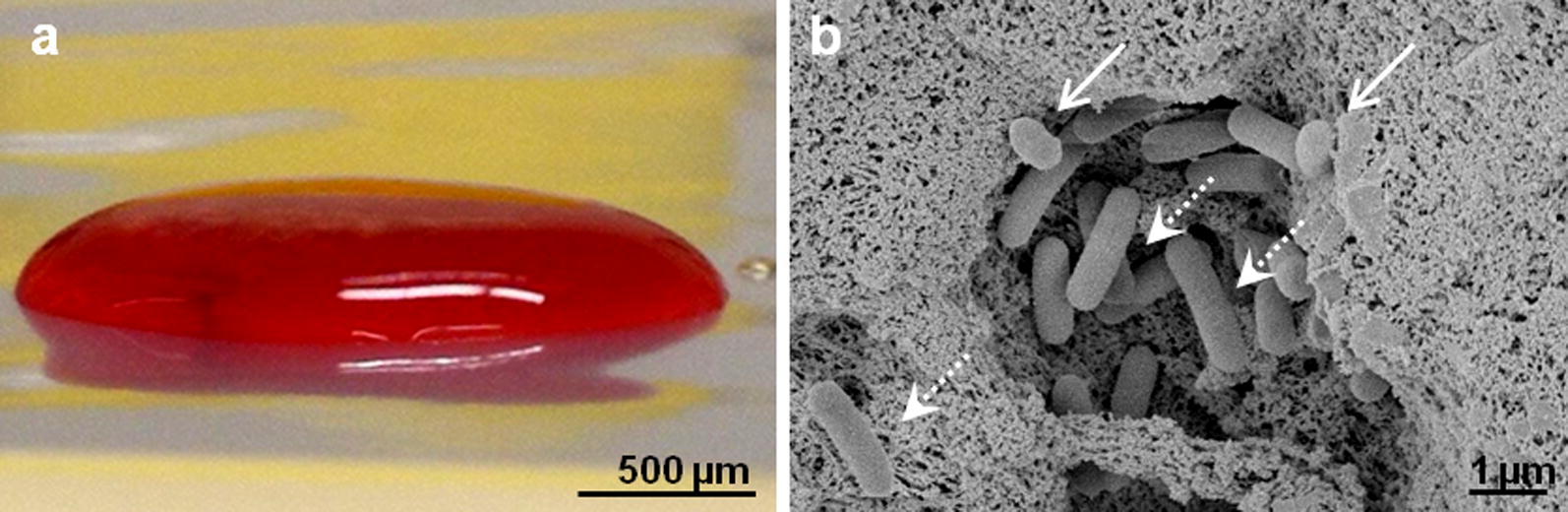
Human plasma biofilm model (hpBIOM). **a** Stable coagula-like disc were generated. **b** By means of SEM, integrated microorganisms attached to the fibrin surface were visible

The hpBIOM was produced by fusion of human plasma and the corresponding buffy coat from the same donor. After the addition of the bacteria and activation of the coagulation cascade, stable coagula-like biofilm discs with integrated pathogens were generated (Fig. 1a). By means of scanning electron microscopy, bacterial colonies were detected on the fibrin scaffold (Fig. 1b). Staining of the glycokalyx revealed the development of the EPS after 1 h.

### Interference of probiotic bacteria with pathogenic species

**Fig. 2:**
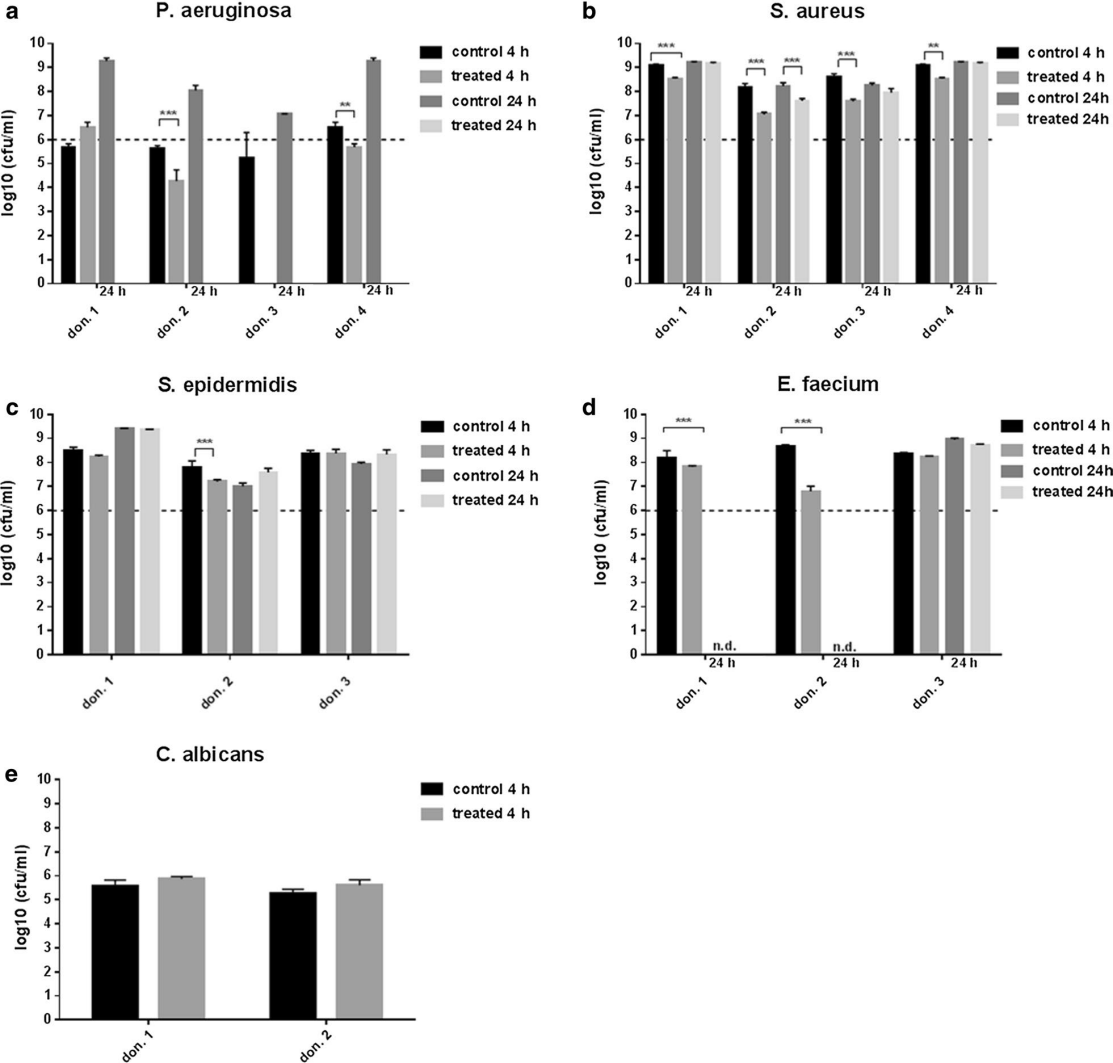
Influence of *L. plantarum* on microbial growth in biofilms. **a** With the exception of two donors, *L. plantarum* successfully eliminated the *P. aeruginosa* infection after 4 h of incubation. After 24 h, *P. aeruginosa* was finally destroyed in biofilms from all donors. **b**–**d** The pathogens *S. aureus*, *S. epidermidis* and *E. faecium* also displayed a slight growth inhibition. The effects are dependent on the individual plasma. **e** The growth rate of *C. albicans* showed no inhibitory influence of *L. plantarum*. don., donor; nd, not determined. All experiments were performed in triplet per donor (*p ≤ 0.05; **p ≤ 0.01; ***p ≤ 0.001)

**Fig. 3:**
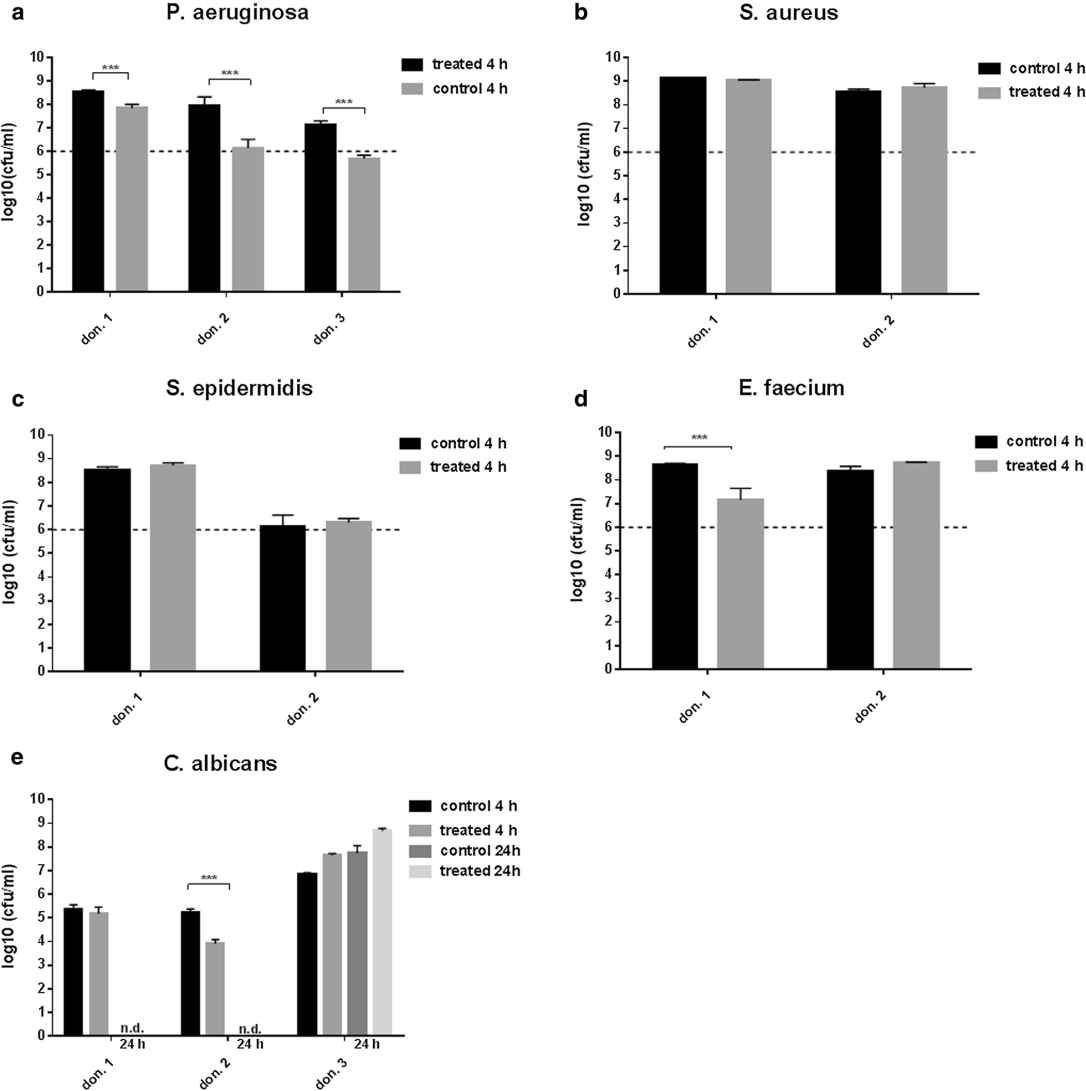
Antimicrobial activity of *B. lactis* in the hpBIOM. **b**, **c** *B. lactis* exerted no inhibitory effects on *S. aureus* and *S. epidermidis*. **a**, **d**, **e** Growth-reducing capacity was determined towards *Pseudomonas*, *E. faecium* and in one donor towards *C. albicans* up to 2 log_10_-reduction rates. The effect was strongly plasma-dependent. don., donor; nd, not determined. All experiments were performed in triplet per donor (*p ≤ 0.05; **p ≤ 0.01; ***p ≤ 0.001)

**Fig. 4:**
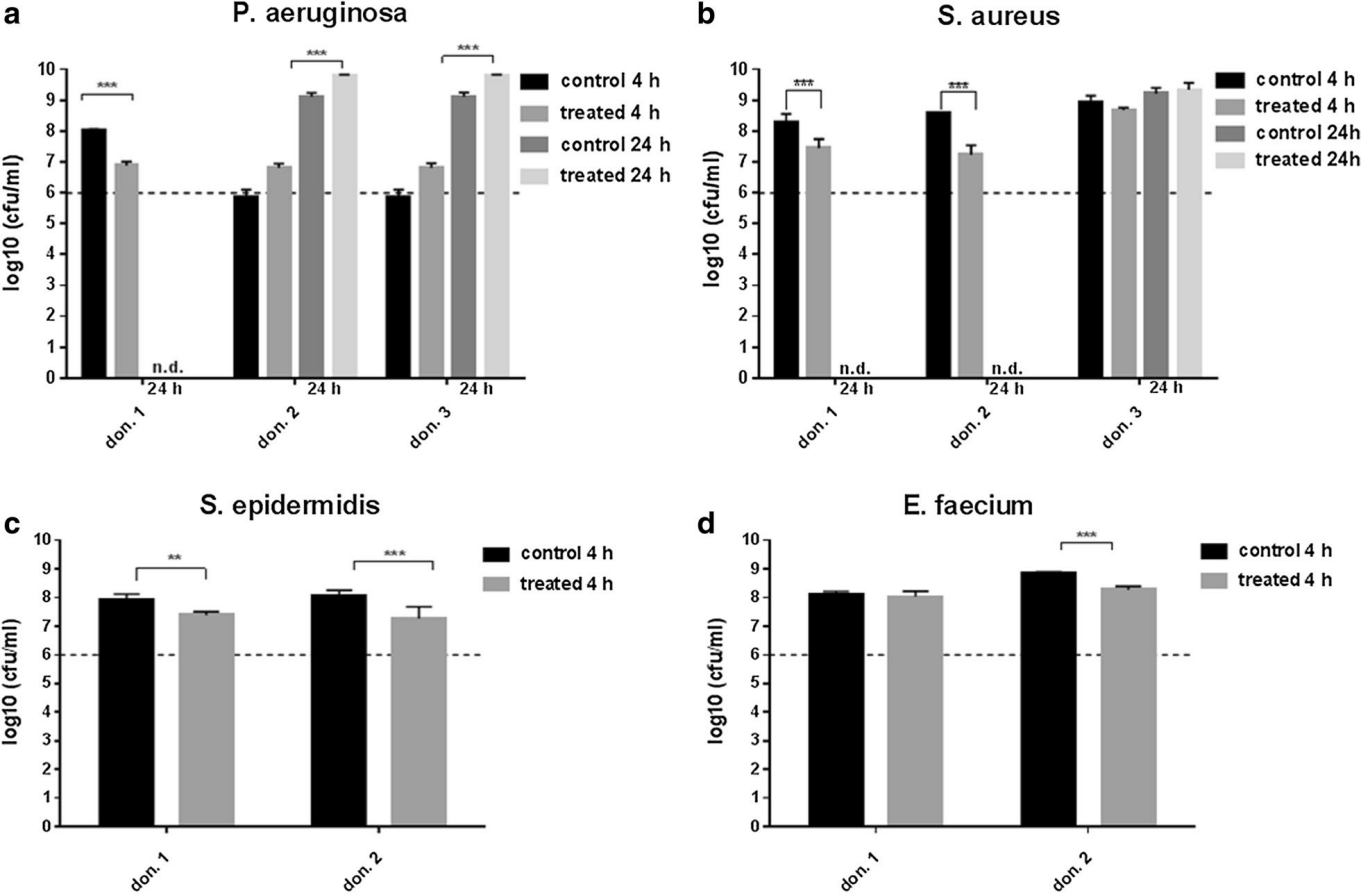
Effect of *S. cerevisiae* on the survival of pathogens in the hpBIOM. **a**, **b** Dependent on the donor, *S. cerevisiae* showed growth-inhibiting capacities towards *P. aeruginosa* and *S. aureus*. **c**,** d** *S. epidermidis* and *E. faecium* were not influenced in a clinically relevant concentration. don., donor; nd, not determined. All experiments were performed in triplet per donor (*p ≤ 0.05; **p ≤ 0.01; ***p ≤ 0.001)

This study involved a systematic analysis of the antimicrobial activity of three probiotics *L. plantarum*, *B. lactis* or *S. cerevisiae* against five clinically relevant pathogens *P. aeruginosa*, *S. aureus*, *S. epidermidis*, *E. faecium* and *C. albicans*. *Lactobacillus plantarum* eliminated the *Pseudomonas* infection after 4 h of incubation, except for biofilms from donor 1 and 2 (Fig. 2a). Finally, after 24 h *P. aeruginosa* was successfully eradicated by *L. plantarum* in hpBIOMs from all donors. No recurrence of the pathogen was detected after 24 h in all plasma probes. The growth of *S. aureus* was also significantly affected in all hpBIOMs by *L. plantarum*, especially after 4 h (Fig. 2b). A log_10_ reduction rate between 0.9–2.1 cfu/ml was detected. In biofilms of plasma from donor 1 and 4, the effect was negated after 24 h. The influence of *L. plantarum* on the growth of *S. epidermidis* displayed variances between the individual donors (Fig. 2c). On the one hand no alteration was observed in hpBIOMs from donor 1 and 3, but, on the other hand, a slight reduction of pathogens was quantified in biofilms from donor 2. The application of *L. plantarum* on biofilms of *E. faecium* resulted in significant inhibition of bacterial growth with a reduction of > 1.8 log_10_ phases. In contrast to the antibacterial effect of *L. plantarum*, no relevant antifungal response was detected towards *C. albicans* (Fig. 2e). *B. lactis* exerted a pathogen-reducing capacity towards *P. aeruginosa* as well as *E. faecium*, while the influence on *E. faecium* growth was strongly donor-specific (Fig. 3a, d). The growth rates of *S. aureus*, *S. epidermidis* and *C. albicans* showed no differences between *B. lactis* treated and non-treated conditions after 4 h of incubation (Fig. 3b, c, e). The application of the yeast *S. cerevisiae* resulted into moderate but significant reduction of the pathogens *S. aureus* and *S. epidermidis* (Fig. 4b, c). The antimicrobial efficiency towards *Pseudomonas* varied in the biofilms. Inhibitory as well as slightly growth-promoting effects were detected (Fig. 4a).

### Combat between *P. aeruginosa* and *L. plantarum*: a presentation via scanning electron microscopy (SEM)

**Fig. 5:**
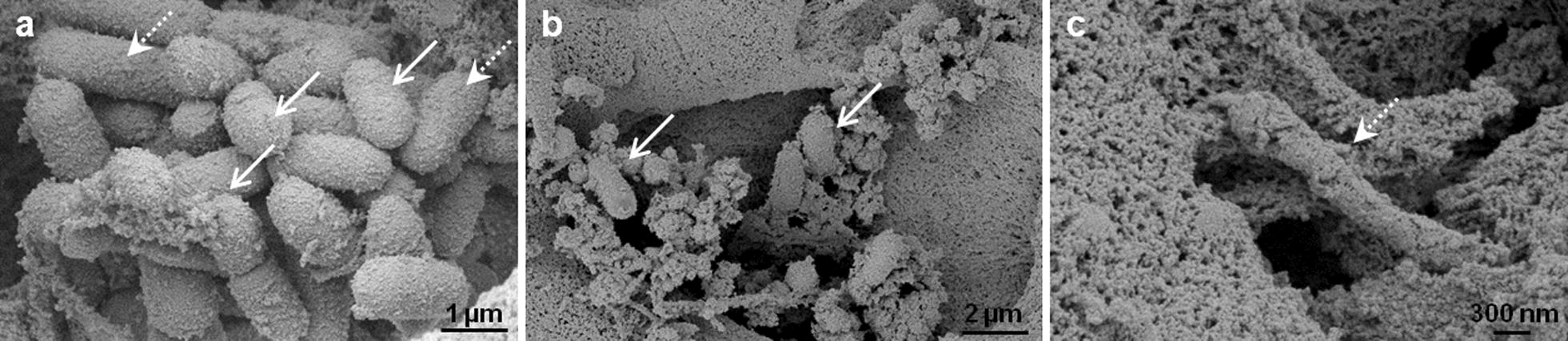
Scanning electron microscopy (SEM) images of a *P. aeruginosa* hpBIOMs. **a** Within the biofilm, bacteria were attached in close vicinity on the surface of the matrix, to develop microcolonies (arrows, dotted lines). **b** One h after supplementation of *L. plantarum*, probiotics arrive the colonies (arrows, straight lines), production of the EPS and scattered interactions were visible. **c** After 4 h, *L. plantarum* has produced a strong glycokalyx, which seemed to cover and subsequently destroy *Pseudomonas*

SEM analysis should give more insight into the organization of *L. plantarum* while eliminating *Pseudomonas* (Fig. 5). During the experiments, *L. plantarum* was applied on top of the biofilm. The eradication process was documented after 1 h and 4 h of incubation. The SEM micrographs illustrated, that *L. plantarum* moved into the hpBIOM and arrived at the *Pseudomonas* colony after 1 h (Fig. 5a arrow, straight lines). The number of *Lactobacilli* increased with time. Scattered probiotic–pathogen interactions were visible (Fig. 5a). *L. plantarum* produced a complex glycokalyx, more rapidly compared to *Pseudomonas* (Fig. 5a, b). This matrix seemed to coat the pathogen, finally, leading to the death of the bacteria (Fig. 5c).

## Discussion

In Europe, at least 5 million people suffer from leg ulcer, as a representative chronic wound. Approximately, 4 million diabetic patients will develop leg or food ulcer within the next 10 years [2]. Patients and their relatives have to bear great misery and the economic load for the health care system is enormous [25].

Non-healing wounds can be considered as a variant of chronic infection. Endogenously, the wound healing process stocks in the inflammatory phase and, externally, chronic wounds are often infected with bacteria and fungi, residing within a self-constructed biomatrix [26]. These biofilms delay wound healing and, due to tremendous survival strategies, are difficult to eliminate.

Various in-vitro biofilm models have been developed for research. The majority consist of bacteria, attached on any adhesive surface. All lack characteristics of the human wound environment and the immune competence. This study used an innovative human plasma biofilm model (hpBIOM), which perfectly mimics a biofilm-challenged human wound milieu. During the initial phases of wound healing, homeostasis and inflammation, vascular permeability increases and blood plasma diffuses into the damaged tissue [27]. Amongst others, the plasma dilutes toxic degradation products, to physically cleaning the wounded area. Further advantage of using human plasma, was the availability of the immune competence for fighting the infection. It contains lymphocytes, granulocytes and monocytes—immune cells that protect the system from infection by microorganisms and decompose damaged cells, resulting in an additional, physiological cleaning effect [28, 29]. The platelets and the complement system in the plasma were utilized to produce coagula-like stable discs, after the administration of pathogenic microorganisms (Fig. 1). Scanning electron microscopy images of *P. aeruginosa* biofilms, stained to the glycokalyx, demonstrated, that the pathogens (as well as probiotics) attached to the fibrin matrix, generate microcolonies and produce EPS (Figs. 1b, 5a–c). Due to all these properties the hpBIOM is postulated to be an appropriate in-vitro biofilm model for translational approach to the clinical situation.

### Bacteriotherapy for eliminating pathogenic microorganisms in biofilms

Health-promoting effects of “good “ lactic acid-producing bacteria were already described centuries ago, especially those belonging to the species *Bifidobacterium* and *Lactobacillus*, by inhibiting the growth of pathogenic bacteria within the colon. Different probiotics are already in use to treat dysbiosis and infections of the gastrointestinal- and urinary tract and dental diseases, e.g. pouchitis [7, 16, 30, 31].

Many studies propose better outcomes after bacteriotherapy by using *L. plantarum*, e.g. in animal models of *P. aeruginosa* infected burn wounds or chronic wounds in diabetic mice. Even a topically applied prophylactic administration of *L. plantarum* induced a health benefit [17, 30, 32]. Some in-vitro studies using surface-attached biofilms, challenged the pathogens to different types of living lactic acid-producing bacteria as well as supernatants or isolated proteins, and confirmed the antimicrobial activity and healing-promoting effects [33–39]. The success was dependent on the applied pathogens and probiotics and their concentrations. However, there is a great need for research addressing the potential of bacteriotherapy and the understanding of the mechanisms in more detail. This study transferred the investigation to the newly established human plasma biofilm model. The selection of pathogenic bacteria was based on the WHO list of priority pathogens for R&D of new antibiotics published in February 2017 [15]. Additionally, a fungal contamination with *C. albicans* was examined.

Plasma preserves from different donors were used for the investigation. The results were not pooled, due to the different immune competences of the donors and the potential influence on the antimicrobial efficiency. In the hpBIOM, it was possible to demonstrate and to confirm the enormous antimicrobial efficiency of *L. plantarum* towards *Pseudomonas* infections (Fig. 2a). By means of SEM, it was possible to visualize the migration into the biofilm and direct pathogen-probiotic interaction (Fig. 5a, b). Furthermore, *L. plantarum* extensively produced a glycokalyx, which seemed to cover and destroy *Pseudomonas* (Fig. 5c). Supplementation of *L. plantarum* to *S. aureus*, *S. epidermidis* and *E. faecium* also induced slight but significant growth reductions (Fig. 2b–d), which was not shown before. The exact mechanisms resulting in the reduction or elimination of these bacteria is currently under investigation in this system. Different possibilities are postulated in other publications. For instance, different lactobacilli species have anti-elastase activity against *P. aeruginosa* [33]. Additionally, the effects of *L. plantarum* were assigned to the secretion of antimicrobial substances, like 4,5-dihydroxy-2,3-pentanedione and 2-methyl-2,3,3,4-tetrahydroxytertahydrofurane, which inhibits quorum sensing [38]. Other antimicrobial substances like hydrogen peroxide, benzoic acid or lactic acid are also secreted by *L. plantarum* [36]. The effect was donor- and time-specific, and thereby considered to be dependent on the immune system of the donor. This thesis was already proved in the gut, where different Bifidobacteria as well as Lactobacilli exerted stimulatory effect on the immune system [16]. This has to be evaluated in progressive studies. Additionally, the constitution of the bacterial cell membrane seems to be a limiting factor, because the highest growth-reducing effects were detected against gram-negative bacteria. The growth rate of *C. albicans* was not affected (Fig. 2e). This species is also surrounded by a strong cell wall. Interestingly, *B. lactis* also exerted a reducing activity towards *Pseudomonas* and *E. faecium* (Fig. 3a, d) and even the yeast *S. cerevisiae* showed slight but significant inhibitory effects on *S. aureus*, *S. epidermidis* and *E. faecium* (Fig. 4b–d). These capacities were not yet determined in human biofilms. Although the reduction of the bacterial burden seemed not to be tremendous in some combinations, it can have major relevance for the wound therapy, because it enhances the chance of reducing bacterial load by the individual immune system. Further tests with a higher number of probiotics or their combinations will be performed, to examine, whether this will improve antimicrobial outcome.

Summarized, this study successfully reproduced a novel human biofilm model. This system still represents an in-vitro model and bares limitations like a time-limited stability or the lack of skin cells. Nevertheless, several improvements were developed compared to current biofilm models. It involves essential factors for analysing biofilms in a translational research approach, namely the individual immune competence and a human wound environment. By means of the hpBIOM, it was possible to systematically screen growth-reducing activity of three probiotics towards five clinically relevant pathogens. It was possible to visualize the elimination process of *L. plantarum* against *P. aeruginosa*. Finally, additional insights into the influence of the probiotic microorganisms *B. lactis* and *S. cerevisiae* could be efficiently obtained. These effects are described for this study design and could be differ after using other concentrations of probiotics or pathogens, respectively. In future studies, the investigation of bacteriotherapy by means of the hpBIOM should be expanded with regard to subcellular and molecular insights. Additionally, the portfolio of probiotics should be increased and in particular, combined therapies of *L. plantarum* and other effective probiotics should be investigated using the hpBIOM.

## Conclusions

A novel human biofilm model, which includes two essential factors for the analysis of biofilms in a translational approach, namely, a human wound milieu and individual human immune competence was reproduced. Especially, the probiotic *L. plantarum* is able to eliminate *P. aeruginosa* and differentially inhibits the growth of the tested pathogens, except of C. albicans. *B. lactis* and *S. cerevisiae* exert growth-inhibiting influence. The antimicrobial activity was strikingly donor-specific.

## Explanation and importance for the field

The presence of biofilms impairs wound healing and reflects one pivotal factor in the development of chronic wounds. Biofilm-growing bacteria display increased antibiotic resistances: Knowledge of the biofilm environment, behavior of the bacteria and novel therapeutic options are prerequisite for improving wound management. Appropriate model systems for the analyses in a direct translational approach are still missing. They lack the human wound milieu and the immune competence. This study used a novel human plasma biofilm model and provides further insights into the efficiency of bacteriotherapy by means of probiotics.

## Data Availability

All data and material are available.

## Abbreviations

B.: *Bifidobacterium*
C.: *Candida*
CSB: Casein-Soy-Bouillon
cfu: colony-forming-unit
don.: donor
E.: Enterococcus
EPS: extracellular polymeric substance
Fig.: figure
h: hour
hpBIOM: human plasma biofilm model
L.: Lactobacillus
MEB: Malt-Extract-Bouillon
Min: minute
MRS: Man–Rogosa–Sharpe
P.: Pseudomonas
rpm: round per minute
SD: standard deviation
SEM: scanning electron microscopy
